# Clinical decision support models for oropharyngeal cancer treatment: design and evaluation of a multi-stage knowledge abstraction and formalization process

**DOI:** 10.1007/s11548-022-02675-3

**Published:** 2022-06-03

**Authors:** Jan Gaebel, Stefanie Mehlhorn, Alexander Oeser, Andreas Dietz, Thomas Neumuth, Matthaeus Stoehr

**Affiliations:** 1 Faculty of Medicine, Innovation Center Computer Assisted Surgery (ICCAS), University of Leipzig, Semmelweisstraße 14, 04103 Leipzig, Germany; 2 Department of Otolaryngology, Head and Neck Surgery, University Hospital Leipzig, Leipzig, Germany

**Keywords:** Clinical decision support systems, Cancer of the oropharynx, Patient-specific modeling, Chemotherapy

## Abstract

**Purpose:**

Treatment decisions in oncology are demanding and affect survival, general health, and quality of life. Expert systems can handle the complexity of the oncological field. We propose the application of a hybrid modeling approach for decision support models consisting of expert-based implementation of a decision model structure and machine-learning (ML) based parameter generation. We demonstrate our approach for the treatment of oropharyngeal cancer.

**Methods:**

We created a clinical decision model based on Bayesian Networks and iteratively optimized its characteristics using structured knowledge engineering approaches. We combined manual adaptation of individual concepts with automatic learning of parameters and causalities. Using data from 94 patient records, we targeted the needed objectivity and clinical significance.

**Results:**

In three iteration steps, we assessed the model with cross-validations. The initial aggregated accuracy of 0.529 could be increased to 0.883 in the final version. The predictive rates of the target nodes range from 0.557 to 0.960.

**Conclusion:**

Combining different methodological approaches requires balancing the complexity of the clinical subject matter with the amount of information available in the dataset for ML application. Our method showed promising results because flaws of one approach can be overcome by the other approach. However, technical integrability as well as clinical acceptance must always be ensured.

## Introduction

High demands are made on treatment decisions in oncology, as they significantly affect survival, general health, and quality of life. Suboptimal diagnostics and treatment decisions may foster serious consequences [1]. To ensure sound decisions that feature reliable results, the medical domain is adapting to interdisciplinary pieces of evidence. They form the foundation of the decision-making process. Expert teams combine both explicit (specialized evidence-based knowledge of guidelines) and implicit knowledge (experience and common knowledge) [2]. Considering the characteristics of the individual patient (e.g., tumor size, comorbidities) and the current treatment expertise, adequate decisions can be made [3, 4].

In the past decades, the development of expert systems (ES) to support physicians in complex decision-making processes was established [5]. Numerous medical models have already been developed with the goal to integrate into ES and reach personalized patient care [6]. Research continues because the expected advantages are apparent: ES utilized on modern computer systems can handle the high complexity, especially of the oncological field. However, several requirements must be met for the successful development and clinical use of an ES:

1. The ES has to depict the patient situation as accurately as possible. The foundation of any ES is a formal representation that allows to demonstrate and analyze the causal dependencies, i.e., clinical decision model [7].
2. The causal relationship existing between information entities must be displayed. Through inference, personalized decisions can be made [8].
3. For sound decisions, those causalities need to be objective and evidence-based [7].
4. ES have to be embedded into existing clinical IT infrastructures. Thus, there should not be any additional effort for physicians with transferring the patient characteristics [9].

In general, to generate clinical decision models (CDM) for expert systems, two methods exist: (1) Expert-based modeling using the manual formalization of medical knowledge or (2) machine learning (ML) algorithms to generate a models’ structure and inference relations [10]. ML algorithms promise to enable a more effective objective approach by deriving patterns from available real-life data [12]. There are, however, also hybrid approaches being used, combining manual expert input and parameter learning using real data sets [32, 33].

The work at hand is an extension of a previous approach to clinical decision models for laryngeal cancer [11]. We have shown that a detailed mathematical translation of disease and patient characteristics in the form of a graphical model, i.e., Bayesian Network, is possible. However, the implementation required an immense amount of development resources. Now, we outline a multi-stage abstracting process as a model creation template. It comprises constructing an expert-based Bayesian Network (BN) structure and ML utilization to integrate causal relations derived from clinical data. This process is demonstrated with the primary treatment for oropharyngeal squamous cell carcinoma (OPSCC).

The worldwide incidence of OPSCC recorded in 2020 was 98,412 cases (79,045 male, 19,367 female), with an age-standardized incidence rate of 1.8 per 100,000 for males and 0.4 per 100,000 for females. It caused 48,143 deaths worldwide (39,590 male, 8,553 female) in 2020 [13]. OPSCC is of increasing scientific interest due to the association with human papillomavirus (HPV) infection, which is responsible for the rising incidence of this entity [14, 15]. Besides tobacco and alcohol consumption, HPV infection, primarily by subtype 16, is described as a risk factor and relevant prognostic factor [16]. In consequence, the eighth edition of the Union for International Cancer Control (UICC) tumor, lymph node, metastasis (TNM) classification (TNM 2017) stages OPSCC on p16-status as a surrogate marker for HPV-driven tumors. In p16-positive OPSCC, nodal staging (*N*) derives from the number of positive neck lymph nodes regardless of extra-nodal extension (ECE) [17]. This change in staging led to a “down-staging” of p16-positive OPSCC. Freitag et al. reported that ECE significantly affected overall and tumor-specific survival in p16-positive patients, although TNM 2017 failed to discriminate overall survival in patients adequately [18]. Nevertheless, the current therapeutic decision for OPSCC is not differentiated based on HPV status, but on the former 7th edition of the TNM classification according to international guidelines [19].

This work contributes to the field of applied medical informatics by.

- Extending an established approach to decision model creation
- Demonstrating the reduction of a model’s complexity but maintaining clinical relevance
- Validating the process of abstraction in clinical models given clinical data

## Methods

We propose a workflow to create a Bayesian Network as a decision model for OPSCC using techniques from knowledge engineering. This comprises (1) the manual creation of the network structure and (2) the calculation of the causal relations with real data. This aims to objectify the causal relationships between the variables using ML. There are multiple stages of this process, which will be described in detail in the following sections:

1. Manually creating the network’s structure with variables depicting clinical concepts
2. Using the data sets to train the networks causal parameters and validating the accuracy of the model
3. Reducing the model’s complexity by clinical feature clarification
4. Reducing the model’s complexity guided by the idea of canonical models [29]

After validating, we adapted the network structure to decrease complexity and simultaneously increase accuracy while keeping the clinical significance and pertinence as high as possible.

### Knowledge engineering

#### Acquisition of knowledge: identifying relevant variables

To transfer knowledge about OPSCC into the knowledge base of the clinical decision model, it was essential to select the explicit knowledge, containing the documentable representations of facts, processes, or experiences [2]. For adequate data sampling and ML-learning processing, the variables need to be recorded in the hospital information system. Thus, a rational selection of realistic variables affecting decisions was vital. This was implemented by reviewing literature and analyzing treatment decision processes in the interdisciplinary tumor board meetings at the University Hospital Leipzig. The following variables were identified as prominent factors for the therapy assessment of OPSCC:

- Tumor extension correlates with prognosis and is therefore taken into account in the staging system TNM 2017 [17].
- The Karnofsky index [23] as the patient’s performance status is the main predictor of response and success of therapies [20], of their comorbidities [21] and quality of life [22].
- Field cancerization in the context of head and neck cancers (HNC) is also a commonly known phenomenon [24]. The detection of a second primary tumor through panendoscopy is very important in the diagnostic process of OPSCC because it is a negative predictor of survival.

Clinical practice guidelines (e.g., NCCN Guidelines [25]) are evidence-based, consensus-driven management references to ensure that all patients receive preventive, diagnostic, treatment, and supportive services that are most likely to lead to optimal outcomes. Based on these guidelines, the treatment options were selected: a) primary surgery, b) systemic/chemotherapy, c) primary radiation, and d) concurrent/adjuvant radiation.

### Clinical cohort data and parameter learning

The medical records of 94 patients diagnosed with OPSCC were collected for analyzing the treatment decision as well as for training the ES. All of them were treated in the University Hospital Leipzig from January 2017 to March 2019. Older records were excluded, because of the update of the staging in TNM 2017 [17]. To be able to calculate and validate the CDM, our clinical advisors extracted the relevant clinical data items from the electronic patient record in an anonymized way. The data were extracted to a CSV file with the names of the columns and values of the patient data matching the names of nodes and states in the BN.

### Knowledge Formalization and Implementation

Among CDM, BN have proven effective as intuitively usable survey tools [26]. A BN represents the conditional dependencies of a set of variables as a directed acyclic graph. It comprises the identified variables (nodes) and causal relations (edges). Nodes exist in different states which describe the possible manifestations of one variable. Conditional probability tables (CPTs) for each node denote conditional dependencies of one state on the occurrence of another [27]. A BN can infer the occurrence of desired target states given evidential data of other variables. The modeling software GeNIe was chosen, which is based on the SMILE framework for structural modeling and inference calculation. The framework also provides a BN learning engine [28].

We abandoned the approach of solely learning the BN, including the network structure and causal dependencies, for two reasons. Firstly, we want to dictate the clinical variables and their causal relations to match the current state of medical knowledge and clinical guidelines, respectively. Depending on the input data, learning the network structure either resulted in too many edges between nodes or in some cases, none at all. Secondly, we wanted to achieve human readability of the CDM. Hence, laying out the nodes and their relations by hand, improved the readability and therefore the utilization of the CDM for further investigation.

After manually creating the BN structure with all clinical entities and their respective manifestations (represented by nodes containing categorial states), we used GeNIe’s inherent parameter learning and validation feature to create the CPTs for each node based. We used a tenfold cross-validation to validate the parameters with the target nodes being the four therapy options.

After evaluation of the results, we applied the following approaches of abstraction to the overall CDM to improve the correctness of the model and re-validate it using the same method.

### Multi-stage abstraction process

Developing the CDM according to literature and guidelines in detail is necessary for sound decisions. But it also implies that several variables exist in a variety of states. This results in high input dimensionality and growing CPTs. Training the CPTs using a sparse dataset led to uncertainty and decreasing accuracy, because some permutations aren’t found in the dataset (e.g., patients with rare characteristics). To increase the coverage of inferencing parameters, either a sufficient amount of data records containing all possible permutations is needed or the structure of the model must be adjusted. The former was not available. It is rarely possible to represent all combinations of variables and states. Therefore, adjustments to the CDM were necessary with assessment of the model from a clinical and a technical/mathematical perspective. Clinically, we built on expert opinions and guideline adherence. Technically, we relied on the idea of canonical models, as proposed by Díez and Druzdzel [29].

### Assessment of clinical significance and clustering of variables

In the first CDM version, we choose the states of the variables describing patient and disease according to the presented situation in tumor board meetings. This resulted in 109 different states and led to a significantly low “density of information” in the validation, see Sect  “Results”. In this step, we analyzed the whole model and identified components that can be simplified without losing clinical significance. To ensure this, we solicited opinions of our current model from clinical experts. In interviews with several ENT experts, we discussed the potential variables that can be aggregated or discarded. We applied proposed changes and presented the new model again to the experts for validation.

Exemplary changes comprised of the primary tumor extent expressed in the T-state of TNM 2017. We analyzed several variables displaying additional tumor extent and contraindications for surgery (e.g., “infiltration carotid artery”). Hence, the variables were condensed to one node expressing the resectability. The variable “N-state” which describes the nodal status existed in eight different states according to the TNM- classification. By experts’ assertion, the knowledge of the nodal details does not influence the treatment option significantly. Hence, to cluster these eight states, we condensed the variable into “unresectable nodal disease”.

The resulting second version of the model contained fewer nodes and edges. The six therapy options remained the same but were condensed into five nodes. The surgical options were condensed into two nodes instead of three. A clinical expert adapted the training data accordingly since the variables changed and the patient data must match the model structure. We re-validated the model, again using a tenfold cross-validation.

### Third iteration and model finalization

**Fig. 1:**
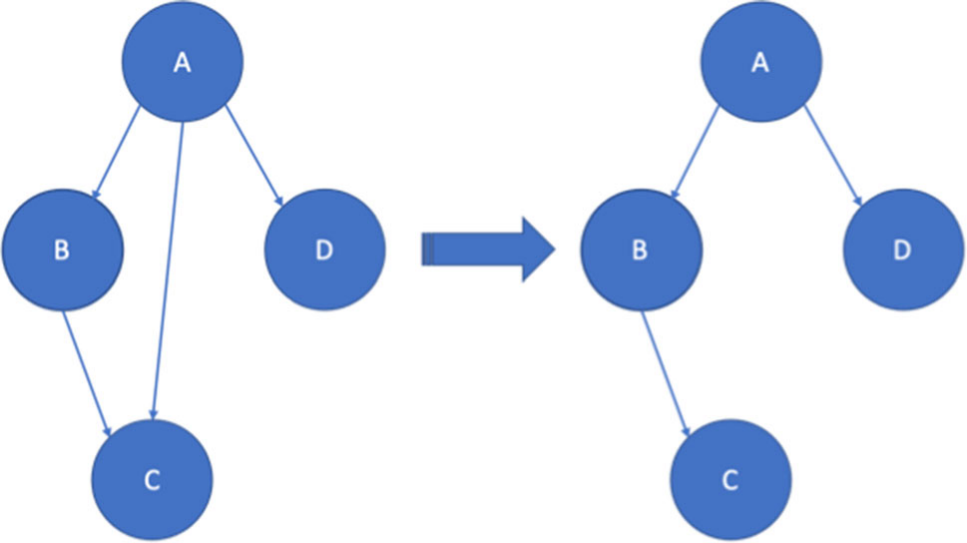
Independence of causal influence

We analyzed the remaining nodes’ states and edges. We applied the independence of causal influence (ICI) principle to our model. ICI implies, that there is no dependency among causal structures, transitivity must be strict and any overleaping dependency must be resolved [29]. Figure 1 displays the simplest form of resolved transitivity. All diagnostic variables and their inferred nodes were made independent from each other, so that for two nodes $X$ and $Y$, there are no links between their parent nodes ${P}_{X}\to {P}_{Y} (with X\ne Y)$. To reduce the complexity further, after identifying relevant parameters, several states were identified as implicit knowledge. These nodes were grouped into one node by applying the leaky ICI principle. The values were discretized into binary pairs of states. The implicit information in these nodes represents mostly information on the general state of health or prognostic factors (e.g., functional limitations after surgery). In regards to clinical practice and expert judgment, we consolidated these states that give only marginal information into the therapy decision.

Again, we validated the resulting model with the same data sets that have been adapted once again to match the updated model structure.

## Results

The first CDM contained six nodes depicting the detailed data on the four therapy options listed in Sect “Knowledge Formalization and Implementation”. Three nodes describe the possibility of surgery in general and then differentiate between neck dissection on both the tumor side and the contralateral side. Two nodes describe the radiation therapy option, as primary therapy and as concurrent. The sixth node depicts the option for chemotherapy. The node for “best supportive care” (meaning the palliative option) was not considered in our evaluation and represents the state if no treatment is possible.

**Fig. 2:**
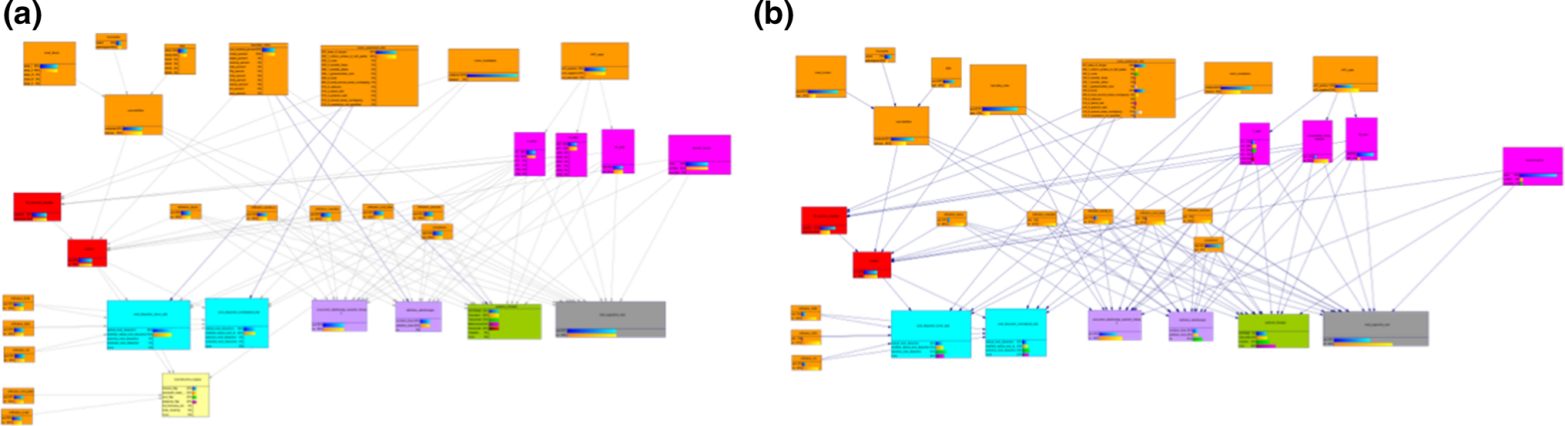
Illustration of **a** first and **b** second version of the OPSCC CDM

As illustrated in Fig. 2, the CDM contains the possible therapy options for OPSCC, shown in the lower part with the cyan, purple, green. The grey node represents best supportive care. All influencing factors (e.g., comorbidities) and tumor characteristics (e.g., TNM) are modeled as parent nodes. Patient-specific data would be propagated through the network to then calculate the most promising therapeutic options. The first version of the model still had quite many dependencies which resulted in a relatively large file size of ~ 15 MB after learning the nodes’ parameters (see Sect“Knowledge Formalization and Implementation”. This is also visible by the number of edges between nodes in Fig. 2. Having this many child-parent-relations with non-binary states results in an exponential growth of the CPT. Hence, the computability of such models is decreased as compared to less complex models.

**Fig. 3:**
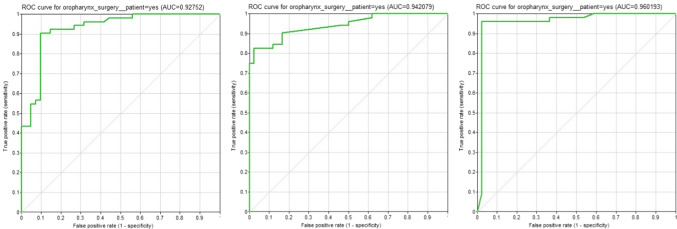
ROC curve for target option “surgery" first all three model versions (first version on the left and the final version on the right)

The accuracy of the first model’s validation resulted in an overall accuracy of 0.529 with 249 correctly predicted individual target variables of 470. However, individual target nodes (e.g., for surgery) differ in their respective accuracy. Figure 3 depicts the ROC curve for the target therapy option “surgery” (expressing the possibility of surgery for a patient). Its first predictive value (left graph) is rather good with an overall accuracy of 0.861. The area under the curve (AUC) is 0.927.

After the first iteration with clinical re-evaluation, the model contained only five target nodes depicting the different therapy options. The cross-validation resulted in an overall accuracy of 0.602 (283 out of 470 correct predictions). The center graph in Fig. 3 depicts again the ROC curve for the surgery node with an AUC = 0.942. The final version of the model was based on canonical models. It contained now only four target nodes, all just expressing Boolean values for the therapy options, i.e., *true* equaling an eligible therapy option and *false* excluding it. The cross-validation resulted in an overall accuracy of 0.883 (332 out of 376 correct predictions). The right graph in Fig. 3 depicts again the ROC curve for the surgery node with an AUC = 0.960.

**Fig. 4:**
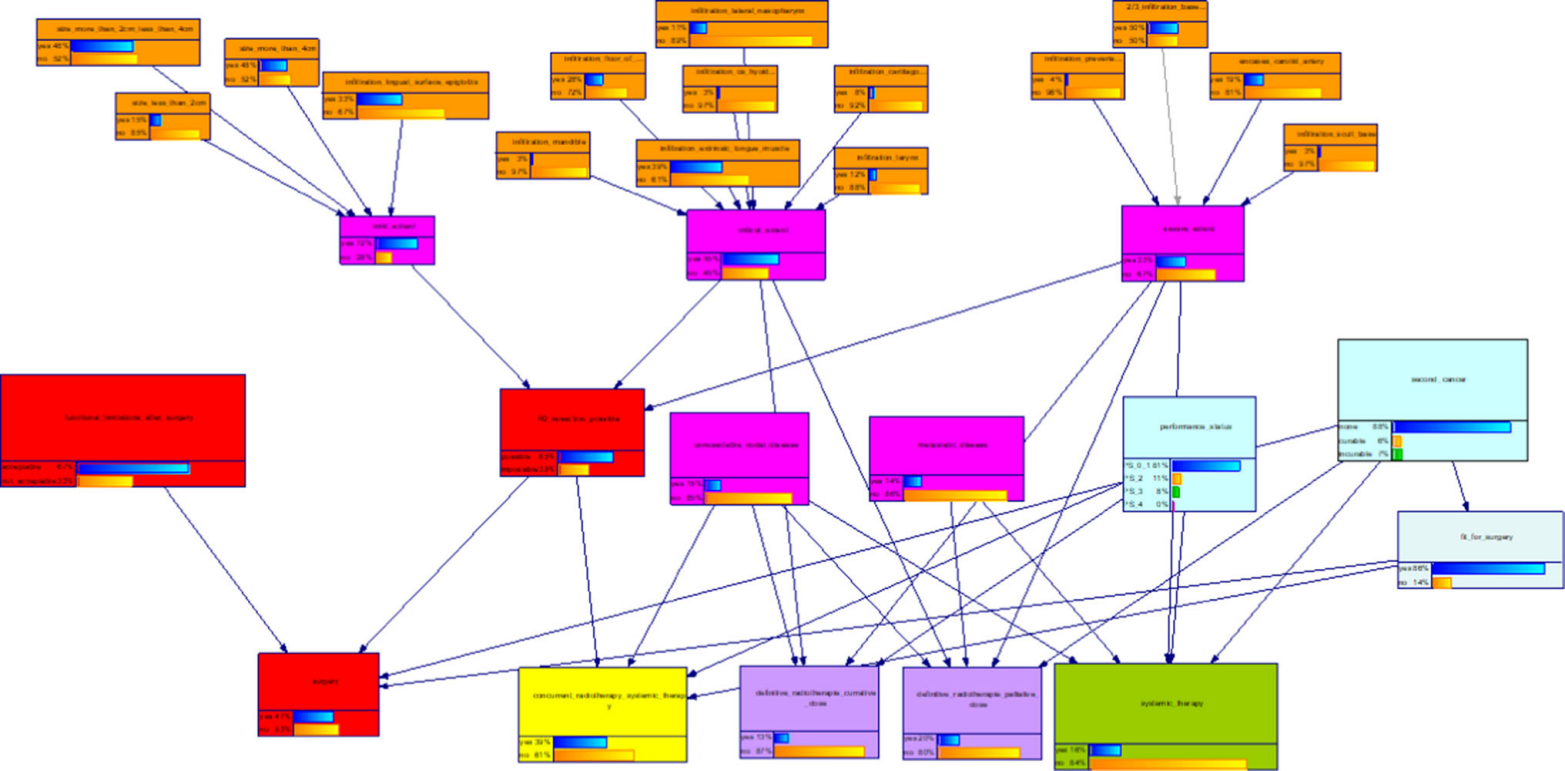
Final version of the OPSCC CDM

**Fig. 5:**
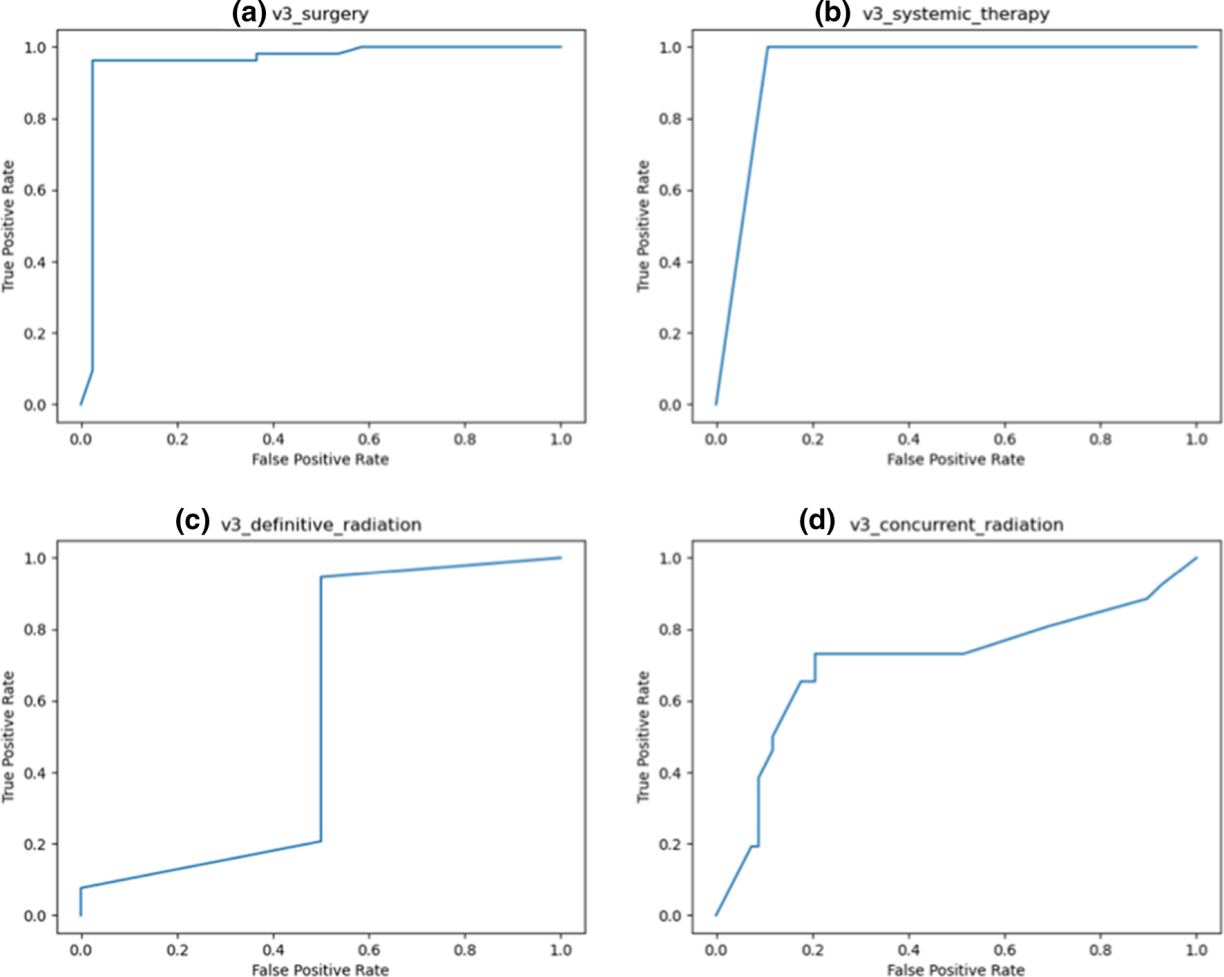
ROC curves for the four therapy nodes. The respective areas under the curve (AUC) are: **a** AUC = 0.960, **b** AUC = 0.946, **c** AUC = 0.557, **d** AUC = 0.708

In its third and final version, illustrated in Fig. 4, the OPSCC CDM contained only Boolean target nodes that represent the therapy options. The surgery node does not differentiate between different surgical options, but rather evaluates general feasibility for surgery. Figure 5 displays the ROC curves for the four therapy options with the respective areas under the curve (AUC).

The results for definitive radiotherapy are rather poor with an AUC of 0.557. The reason is that in the test data, there are only two patients who received primary radiotherapy. Both patients were falsely predicted.

## Discussion

Based on clinical practice guidelines and evaluation of treatment decisions of OPSCC in the tumor board, we could formalize therapeutic knowledge explicitly in a Bayesian Network. Knowledge engineering allowed to represent expert knowledge in this format systematically. The combination of expert-based modeling of the network and calculating the inference parameters using ML algorithms can only be applied if the complexity of the network and the size of the dataset are balanced. A limited coverage of information, as it existed in our first model does not lead to valid representations. The main problem was the exponential growth of the CPTs depending on the number of parent nodes and states influenced our work with defining and learning the parameters. Adapting the network and dataset was necessary to increase the accuracy. However, in current clinical practice, without supportive preprocessing, the hospital information system cannot provide enough structured and detailed training datasets to ensure a realistic depiction of the desired situation. Furthermore, the ML-based approach to structural learning aims to find an optimal representation of the given data which does not necessarily correspond to the clinical guidelines and thus prevents the resulting models from application in clinical routine.

We proposed an approach to structured reduction of complexity of a CDM. The first step was to reduce the model from a clinical perspective. Together with clinical experts, we identified variables that could be removed or aggregated. We approached this similar to the stepwise backwards elimination principle from dimensionality reduction [30]. This helped us create a slimmer model that still contained all relevant clinical concepts. However, further analysis was necessary. Hence, we relied on mathematical approaches, i.e., canonical models [29]. We applied ICI model structures. They were, however, not purely implemented in our CDM, since a strict reshaping of the model’s structure would have resulted in a decreased human readability. For clinical application, next to clinical relevance and accuracy, we also aimed for physicians’ acceptance. Hence, readability and comprehensibility of a CDM must be regarded. However, we were able to find practical tools within the canonical models that strengthened our model and increase its accuracy.

From a cognitive science point of view, expert problem-solving abilities differ from non-expert capabilities. Experts can consider symptoms, diagnoses, differential diagnostic problems, and therapy decisions within a small framework of decision-relevant facts (small world hypothesis) [31]. They relate these parameters (“is a necessary condition for”, “is an optional condition for” or “is an exclusion criterion for”) and thus keep the hypothesis section small by selecting specific information. Newcomers in a field (e.g., assistant physician) tended to increase the size of hypothesis-section, which lead to difficult decision-making. We aimed to implement a CDM that would cover both perspectives. However, the technical restrictions that forced us to revise the initial model. We argue that this helped us depicting the experts’ views inside the formal model to help novel users understand the structured and decision-relevant thinking. Despite considering various influencing factors, a CDM provides only general statements and is never fully adapted to the individual case. Therefore, a comparison with the opinion of experienced physicians should never be dispensed when using such expert systems. We aimed to provide a tool for the potential study of a patient’s case with a transparent representation and comprehensible causality depiction.

## Conclusion

We developed a CDM for treatment decision support of OPSCC based on BN. Our intent was the formalization and clinically relevant representation of primary treatment of OPSCC. We identified complex structures inside the model and adapted with formal strategies without compromising clinical significance. Using a small clinical data set, we demonstrated the capabilities of parameter training in a manually built CDM. We validated the method of the clinical subject matter with the amount of information available in the data set for ML application.
